# Effect of long-term irrigation patterns on phosphorus forms and distribution in the brown soil zone

**DOI:** 10.1371/journal.pone.0188361

**Published:** 2017-11-20

**Authors:** Chang Liu, Xiuli Dang, Melanie A. Mayes, Leilei Chen, Yulong Zhang

**Affiliations:** 1 College of Land and Environment, Shenyang Agricultural University, Shenyang, Liaoning, China; 2 Oak Ridge National Laboratory, Environmental Sciences Division, Climate Change Science Institute, Oak Ridge, Tennessee, United States of America; 3 Integrated Technology Department, Chifeng Environmental Monitoring Central Station, Chifeng, Inner Mongolia, China; ICAR Research Complex for NEH Region, INDIA

## Abstract

Continuous application of P fertilizers under different irrigation patterns can change soil phosphorus (P) chemical behavior and increase soil P levels that are of environmental concern. To assess the effect of long-term different irrigation patterns on soil P fractions and availability, this study examined sequential changes in soil organic P and inorganic P from furrow irrigation (FI), surface drip irrigation (SUR), and subsurface drip irrigation (SDI) in the brown soil zone (0–60 cm) during 1998 to 2011. Analyses of soil P behavior showed that the levels of total P are frequently high on top soil layers. The total P (TP) contents of the entire soil profiles under three irrigation treatments were 830.2–3180.1 mg/kg. The contents of available P (AP) were 72.6–319.3 mg P/kg soil through soil profiles. The greatest TP and AP contents were obtained within the upper soil layers in FI. Results of Hedley’s P fractionation indicate that HCl-P is a dominant form and the proportion to TP ranges from 29% to 43% in all three methods. The contents of various fractions of P were positively correlated with the levels of total carbon (TC), total inorganic carbon (TIC), and calcium (Ca), whereas the P fractions had negative correlation with pH in all soil samples. Regression models proved that NaHCO_3_-P_o_ was an important factor in determining the amount of AP in FI. H_2_O-P_o_, NaHCO_3_-P_o_, and NaOH-P_i_ were related to available P values in SUR. NaHCO_3_-P_o_ and NaOH-P_o_ played important roles in SDI. The tomato yield under SUR was higher than SDI and FI. The difference of P availability was also controlled by the physicochemical soil properties under different irrigation schedule. SUR was a reasonable irrigation pattern to improve the utilization efficiency of water and fertilizer.

## Introduction

Phosphorus (P) is regarded as one of the essential elements for plant growth and development, since it plays a key role in plant metabolism and energy transformation [1]. Therefore, P is added to cultivated soils worldwide through chemical or organic (e.g., manure and sewage sludge) fertilization. However, continuous application of P fertilizers in excess of crop demand poses an increasing risk of P loss from agricultural soil through runoff and leaching to surface water and groundwater [2–5]. A peak in global P production is predicted to occur in the next decades. Thus, the P dynamics in soils and cycling in agro-ecosystems have received increasing interest by international researchers [6–8].

P is mainly present in both organic and inorganic forms in soil, and various fractions could be further sequentially extracted from inorganic P based on their different binding abilities [9]. However, only a small fraction of total soil P is in a form directly available for microbial or plant uptake [10]. Organic P was the primary available P source for plant on a non-fertilized soil [11–12]. Water-soluble P and NaHCO_3_-extractable P were considered more labile P fractions [13].

Many P fractions can be transported into surface water [14–15] by irrigation or precipitation. The plants require an adequate amount of soil moisture for their growth [14]. However, the soil can only store a limited amount of water and only a part of this storage is available to the plant. For this reason, many irrigation technologies have been developed to reduce water loss and enhance efficiency of applied water in irrigated agriculture [14], including furrow irrigation, sprinkle irrigation, drip irrigation, surface irrigation, and subsurface irrigation [16]. According to the statistics, the area of irrigation was up to 324 Mha worldwide in 2012, about 49% of which occurred in India, China and USA [17]. Furrow irrigation was the most common method of agricultural irrigation used worldwide, because it was simple and cheap. Whereas, a reliable and suitable irrigation can improve irrigation efficiency and crop production [18]. Alam [19] reported that subsurface irrigation and drip irrigation can potentially decrease water using by around 30–40% compared to furrow irrigation in Kansas, USA [14]. Therefore, some low volume irrigation systems (bubblers, micro and drip) greatly increase the irrigation efficiency by delivering precise amount of water directly to the root of plant, which substantially eliminates water waste and runoff.

Several studies have examined the effects of irrigation pattern on P levels and P fractions in soils. Silber [20] and Ben-Gal [21] pointed that high irrigation frequency also increased P uptake and affect P distribution and mobility into the soil profile. Sharpley [22] reported that an improper irrigation management can induce soil P surface runoff. Condron [23] observed that long-term flood irrigation increased P transfer through soil profiles by leaching in coarse-textured and stony soils. Ahmed [15] found that the sprinkler irrigation can release significant amounts of P to surface water or groundwater. Vertical distribution and plant availability of soil P under subsurface irrigation were investigated in a five-year tomato greenhouse experiment by Wang [24], which suggested that the greater P availability for plant uptake occurred under the irrigation of relatively high frequency and low water quantity of each irrigation event. A number of studies have shown that relatively high-water content under frequent irrigation leads to a greater P mobility and availability [25–27], which also improves the P conversion in the internal part of plant by enhancing root–shoot ratio and root elongation by releasing organic acids, protons [28], or phosphatase enzymes to effectively obtain P [29].

Numerous studies have been carried out to investigate the distribution and transformation of P fractions in irrigated agricultural area for short term. Few studies were focused on the impact of P fractions distribution and transformation under different irrigation patterns in greenhouse, since irrigation is the only way to supply water to greenhouse-grown plants in winter growing season. Greenhouse is widely used to maintain a controlled environment for optimal crop production and maximal profits [30], especially in China. China is the country with the largest area (2 million hectares) of greenhouse all over the world [31]. Therefore, developing more efficient irrigation patterns to mitigate water scarcity, reduce P loss from soil and increase crop yields in greenhouse is critical. Yang [32] investigated total P distribution throughout soil depth under different irrigation patterns in greenhouse, and the results revealed that both organic P and inorganic P were significantly affected by the irrigation systems. Nevertheless, this study has not reached firm conclusions on the relationship between the complexity of soil physicochemical properties and P fractions behavior. NaOH-P is the P bound to metal oxides (aluminum and iron), and HCl-P represents the P associated with calcium and magnesium [33]. NaHCO_3_-P_i_ and NaOH-P_i_ were significantly positively correlated with Al and Fe contents but negatively correlated with content of organic matter (OM) [34]. 77% to 98% of total P was associated with the contents of OM and Fe oxides [33]. Thus, it is necessary to examine the effects of irrigation patterns on P fractions availability, as well as the impact factors of P transformation in greenhouse.

In present, the tendency of excessive P fertilizer application is still increasing in greenhouse. Excessive P fertilizer application resulted in P accumulation in soils, and P leaching into groundwater. Therefore, P immobilization or mobilization mechanisms involved is important to decline the risk of P leaching in soils. Despite substantial studies of P levels and P fractions with fertilization were reported on different soils in China. However, little is known about the sequentially extractable P fractionations on profile samples under long-term different irrigation patterns in greenhouse. Different irrigation patterns might affect P transfers and forms. Further research effort on optimizing irrigation pattern to improve greenhouse sustainability and protect water quality is therefore warranted. The objectives of this paper were: 1) to determine general differences in P fractions influenced by different irrigation patterns from greenhouse soils. 2) to determine the relationship of the P fractions and basic soil properties.

## Materials and methods

### Study sites and soil

The study sites were located at the Experimental Station of the Shenyang Agricultural University in Shenyang, northeast China (41°31′N, 123°24′E, 51.6 m above sea level). Annual mean precipitation in the area is 720 mm. Mean summer air temperature is 24°C, and mean winter air temperature is −9.2 C. Soil is classified as a brown soil (Mollic Gleysols in the FAO–UNESCO system), with high organic matter and soil viscosity-density [35]. pH 6.80; organic C 22.7 g/kg; total P 1.86 g/kg, total N 1.30 g/kg, total K 17.60 g/kg; available P 103.10 mg/kg, available N 96.91mg/kg, available K 164.00 mg/kg.

### Experimental design

The long-term irrigation experiment in the greenhouse was conducted from 1998 to 2011. The experiment was arranged in a randomized complete block design with four replicates. Forty-day-old tomato seedlings were transplanted at 30 cm within the row and 50 cm between rows on May 6 and harvested in August 16. Treatments included furrow irrigation (FI), surface drip irrigation (SUR) and Subsurface drip irrigation (SDI). Each treatment had four plots, with an area of 8.25 m^2^ each. Plastic films were vertically placed to a depth of 100 cm to avoid leakage of water and nutrient between the adjacent plots. All treatments were applied with urea 300 kg/hm^2^, diammonium phosphate 225 kg/hm^2^, potassium sulfate 300 kg/hm^2^, and poultry manure each year. Poultry manure (37.5*10^3^ kg/ha) was applied to the soil surface and later incorporate into 20–30 cm soil layer under soil surface by means of manual ploughing 5 days before transplanting the seedlings. Urea (300 kg/ha), di-ammonium phosphate [(NH_4_)_2_HPO_4_] (225 kg/ha), and potassium sulfate [K_2_SO_4_] (300 kg/ha) were band-applied as basal dressing in the row at a depth of 20–30 cm when planted. Urea was topdressed twice (150 kg/ha for each) during the period of fruit expansion.

In the FI pattern, the irrigation pipe with an interior diameter of 4–6 cm was used to supply water. In SUR, drip pipes were placed on soil surface along the row, with 5 cm distance from the plants. Each plant had one dripper, with a discharge rate of 2 L/h. The pipes used in this experiment were made of black polyethylene with one micropore every 30 cm (Jiyuan Irrigation Co., China). In SDI, the pipes same as SUR were placed at 0.3 m below soil surface.

### Irrigation amount measure

Ceramic tensiometers (Soil Moisture Equipment Co., Santa Barbara, USA) were placed vertically at soil depths of 10, 20, 30, 40 and 50 cm adjacent to the irrigation pipes in the middle of each plot to monitor soil water potential every day manually. Initial irrigation began when soil water potential reached 40 kPa (60% of field capacity), which was showed by ceramic tensiometers at 20 cm depth. Irrigation stopped when soil water potential reached 6 kPa, which equaled field capacity at those points. The upper and lower limits were 40 and 6 kPa, respectively, for all treatments. Irrigation amount was calculated by the equation:

$$Q=\left(W_{u}-W_{d}\right)\times H\times a\times 10000$$

where *Q* is the irrigation amount (m^3^ ha^−1^); *W*_*u*_ (cm^3^ cm^−3^) is the soil water content at 6 kpa of soil water potential; *W*_*d*_ (cm^3^ cm^−3^) is the soil water content at 40 kpa of soil water potential; *H* (m) is the soil wetted depth (0.3 m for SI and 0.4 m for FI and DI, respectively); *a* is the soil wetness coefficient (1.0 for FI and 0.5 for DI and SI, respectively). The irrigation frequency was 6, 11, and 12 times during tomato growth season for FI, SUR, and SDI, respectively. Irrigation amount per event was 383 m^3^/ha for FI and 174 m^3^/ha and 141 m^3^/ha for SUR and SDI, respectively. Total irrigation amounts for FI, SUR, and SDI were 2295.9, 1912.7, and 1691.9 m^3^/ha, respectively.

### Soil sampling and analysis

Soil cores were collected using soil auger (Eijkelkamp) at five different depths: 0–10, 10–20, 20–30, 30–40, and 40–60 cm from each experimental plot in late July 2011. These samples were air-dried and screened through a 2-mm sieve and stored at 4°C in glass bottles until they were analyzed.

The pH value of each soil sample was measured in a 1:10 solid/liquid ratio suspension using a combination pH electrode. Soil total carbon (TC) was determined by element analyzer (Elementer Vario EL III, Germany). Total inorganic carbon (TIC) was removed from 0.5 g soil samples using a 3 M HCl extraction for 24 h, enabling measurement of total organic carbon (TOC) in the residue [36]. The difference between TC and TOC was considered as TIC. To determine oxalate extractable iron (Fe_ox_) and aluminum (Al_ox_), the soil samples were shaken in the dark with an acid (pH 3.0) ammonium oxalate buffer solution (0.2 M, 1/25 dry weight per volume) for 4 h and filtered with paper filters [37]. The content of Fe_ox_ and Al_ox_ were measured using ICP-MS (Varian MPX, USA). The exchangeable calcium (Ca^2+^) was extracted with 1 mol/L ammonium oxalate (pH 7.0, soil: extractant ratio 1:25) for 0.5 h and measured using ICP-MS (Varian MPX, USA).

Total P (TP) content of the soil was determined by molybdenum blue colorimetric method after digestion with 1 mol/L H_2_SO_4_ and HClO_4_ [38]. Available P (AP) was determined by using a soil to solution (0.5 mol/L NaHCO_3_) ratio 1:20 and 30 min of shaking [39].

Fractionations of soil P were sequentially extracted according to the modified Hedley’s fractionation [11]. Extraction with water was the first step of the procedure. A total of 0.5 g of soil was added into centrifuge tubes, and deionized water was added to bring the final liquid volume to 30 mL. The solution was then shaken on an orbital shaker at 200 rpm for 16 h. The extract was centrifuged at 4000 rpm for 10 min at room temperature (22°C) and filtered through a 0.45 μm cellulose filter. H_2_O-P_i_ was determined from this initial extract, and a portion of the filtrate was autoclaved with H_2_SO_4_ and ammonium persulfate at 103.4 kPa and 121°C for 1 h to determine H_2_O-P. H_2_O-P_o_ was calculated as the difference between H_2_O-P and H_2_O-P_i_. The extraction procedure described above was repeated with 0.5 M NaHCO_3_ (pH 8.5), followed by 0.1 M NaOH. The residue from the following sequential extractions was extracted by 1 M HCl, and HCl-P was measured. Each of these steps was used at the same shaking time for 16 h, and phosphate was determined by the molydate colorimetric method [38]. For the last step, the residues left of the fractionation (HCl) were transferred to heat- and acid-resistant tubes, digested at 360°C for 3 h with concentrated H_2_SO_4_ and H_2_O_2_ [39].

### Statistical analysis

One-way ANOVA combined with Duncan’s multiple range tests were used to determine differences among the treatments. Statistical significance was assigned at the P≤0.05 level.

## Results and discussion

### Influence of different irrigation patterns on soil properties

The change in soil properties was a result of the long-term different irrigation patterns, since the initial soil properties of three irrigation patterns were the same (Table 1). The change in TC contents in all soil samples ranged from 7.9 to 19.18 g/kg. Lower TC value (16.13 g/kg) was observed under SDI in 0–20 cm, whereas TP value was found higher under SDI than FI and SUR down to 60 cm. This mostly resulted from the different irrigation amount and irrigation position. In this study, the organic fertilizers were applied on surface layer, and nutrients were vertically transported with irrigation water through penetration, evaporation and other complex chemical reactions in the soil. In topsoil layer, the amount under SDI was less than FI and SUR, which resulted in a higher organic matter decomposition rate under low soil moisture and rich aeration conditions in surface horizons. However, at the depth of 20–60 cm, the soil moisture under SDI was more than other two treatments, since the SDI irrigation tubes were placed in subsurface soil, which could restrict the decomposition of organic matter, and enhance the TC contents in this layer. Table 1 displays that the TIC and TOC contents slightly diminished as the depth increased from 0 to 60 cm. In the 0–60 cm layer, the contents of Fe_ox_ decreased with increasing soil depth under FI, SUR, and SDI. Fe_ox_ contents under SUR and SDI were significantly higher than that of FI. Fe oxalate represents non-crystalline Fe oxides [40–41] whose bioreduction rates are controlled by oxide surface area and thermodynamic solubility. Among all three irrigation treatments, the content of Fe_ox_ was supposed to be higher under FI than the other two, due to the greater irrigation volume and poorer soil aeration under FI. However, Fe_ox_ concentration exhibited inverse trends in SUR and SDI, since Fe_ox_ value is enhanced under alternate soil drying and wetting cycles, and irrigation frequency under SUR and SDI were higher than that under FI. The significant difference of Fe_ox_ value was observed in the 0–20 cm layer under all treatments. The Al_ox_ distribution exhibited similar pattern as Fe_ox_ through the profile. The Al_ox_ concentration continually decreased with increasing soil depth, and its contents followed the order of SDI> SUR> FI at depths of 0–60 cm as shown in Table 1. The contents of Ca were generally higher in upper soil layers and decreased with increasing soil depth with the trend of FI>SDI>SUR, and significant differences were observed among the treatments for equivalent depth ranging from 0–30 cm. The major reason was that the one-event irrigation amount and evaporation rate of FI were higher than those of SUR and SDI. Therefore, the salt ions could be transferred to the soil surface from the subsoil layer. The soil was wetted partially when SUR was applied, and the evaporation intensity and soil moisture movement rate were lower than those under FI. In addition, the Ca accumulating on the soil surface could move down by leaching when next irrigation event occurred. The upward movement of soil water seemed to dominate above the irrigated pipe. Therefore, Ca was transferred to the soil surface and accumulated. The pH continually increased with increasing soil depth, which may be a result of the decomposition of soil organic matter and acidic ions (H^+^, Al^3+,^ NO_3_^-^) transported to upper soil through evaporation reaction. Whereas, no significant difference was found among the three irrigation patterns within each soil layer.

**Table 1:** Soil properties under three irrigation patterns.

Depth	Methods	TC	TOC	TIC	Fe_ox_	Al_ox_	Ca^2+^	pH
(cm)		(g/kg)	(g/kg)	(g/kg)	(mg/kg)	(mg/kg)	(mg/kg)	
	**FI**^A^	18±1.21a	2.3±0.12a	15.6±1.31a	30.5±0.2c	19.4±0.5b	2378±31a	6.4±0.1a
**0**–**10**	**SUR**^B^	19.18 ±0.82a	5.9±0.29b	13.3±0.65a	79.9±14.2b	23.9±3.3ab	1711±40.5b	6.4 a
	**SDI**^C^	16.13±0.36ab	3.1±0.68a	13±0.62a	90.5±7.8a	28.4±0.8a	2128±40.8c	6.4 a
	**FI**	14.39±0.47a	2.4±0.17a	12±0.58a	30.2±0.2c	18.5±0.1b	1285±27.1b	6.8±0.2a
**10**–**20**	**SUR**	15.74±0.57b	5.2±0.38b	10.6±0.19a	76.7±12.0b	22.9±3.0b	986±36.7c	6.6±0.1a
	**SDI**	14.27±0.75a	3.2±0.75a	11.1±1.34a	99.6±7.9a	29.0±0.8a	1406±13.9a	6.7±0.2a
	**FI**	11±0.48a	2.2±0.06a	8.8±0.46a	25.3±0.8b	14.6±0.4b	535±42.2a	7.0±0.1a
**20**–**30**	**SUR**	13.01±1a	4.8±0.25b	8.3±0.76a	78.9±12.8a	22.3±3.7a	347±38.2b	6.8±0.2a
	**SDI**	13.53±0.68a	3.3±0.88ab	10.3±1.36a	99.1±4.3a	25.5±0.1a	401±9.5b	6.8±0.1a
	**FI**	8.36±0.21a	2.2±0.08a	6.2±0.3a	24.3±1.8b	13.7±0.6b	260±35.4a	7.0±0.2a
**30**–**40**	**SUR**	9.76±0.91a	4.7±0.22b	5.1±0.69a	71.8±11.0b	19.3±2.8a	234±26.0a	6.9±0.1a
	**SDI**	10.53±0.21a	4.2±1.9b	6.3±2.1a	80.8±0.6a	22.2±1.0a	254±21.4a	7.1±0.1a
	**FI**	7.9±0.58a	2.2±0.02a	5.7±0.57a	21.2±0.4b	14.9±0.2b	245±10.6a	7.0±0.2a
**40**–**60**	**SUR**	8.45±0.57a	4.5±0.03b	3.9±0.53a	63.7±7.8b	18.4±1.8ab	179±9.4a	7.1±0.1a
	**SDI**	9.58±0.51a	3.22±0.68ab	6.4±1.1a	77.2±0.9a	21.9±1.8a	205±16.8a	7.1±0.2a

Means ± standard errors followed different lowercase letters denote significant (P <0.05) differences among the treatments in a given soil horizon.

A furrow irrigation.

B surface drip irrigation.

C subsurface drip irrigation.

### Influence of different irrigation patterns on soil P distribution

#### Total P

**Fig 1:**
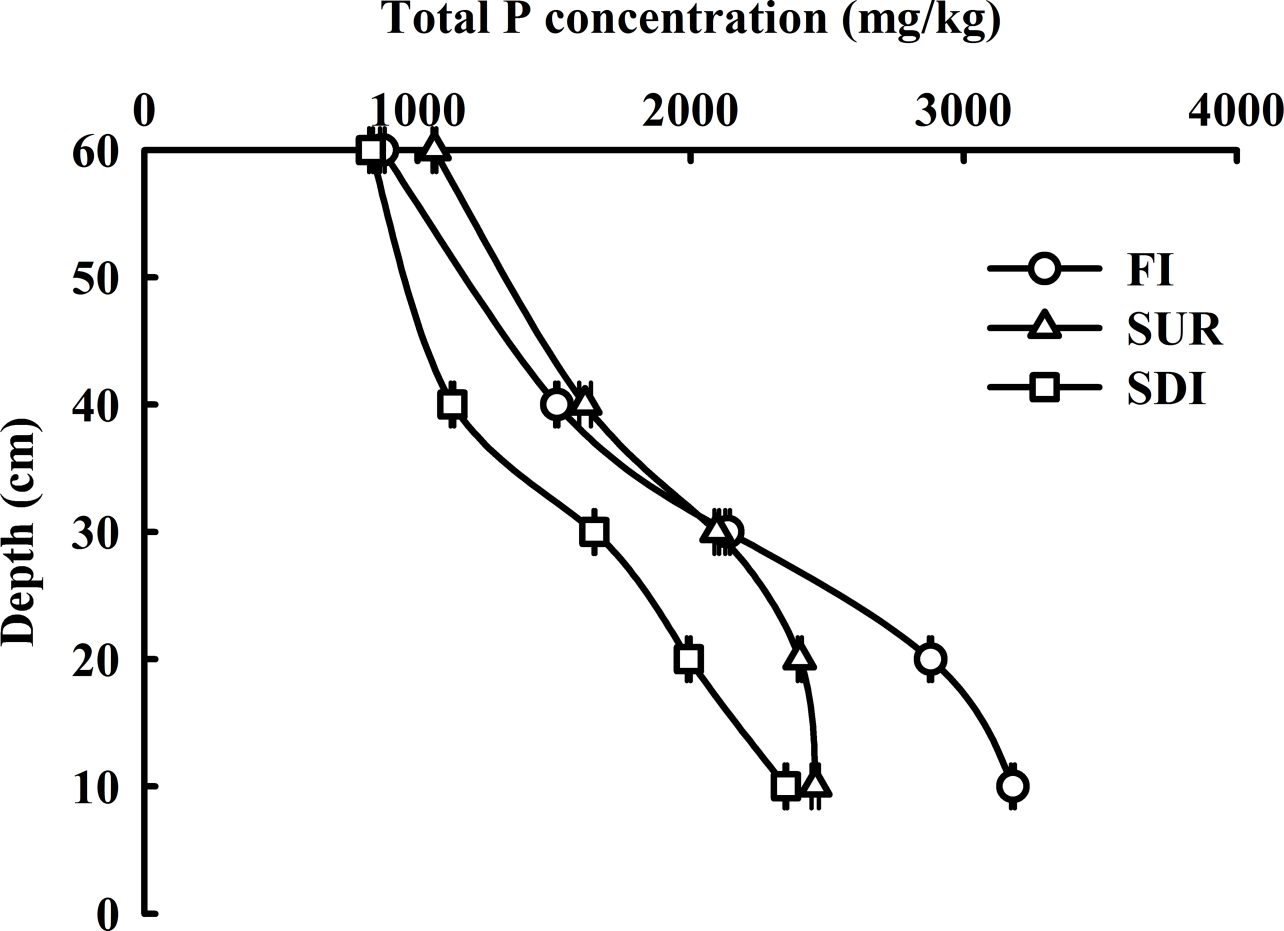
TP distribution in soil profile (0–60 cm).

The changes in the level of TP at different soil depths under FI, SUR, and SDI are shown in Fig 1. The range of TP contents were 871–3180 mg P/kg, 1062–2456 mg P/kg and 830–2348 mg P/kg soil at the depths of 0 to 60 cm under FI, SUR and SDI, respectively. Generally, TP continually decreased with increasing soil depth under all three irrigation patterns.

**Fig 2:**
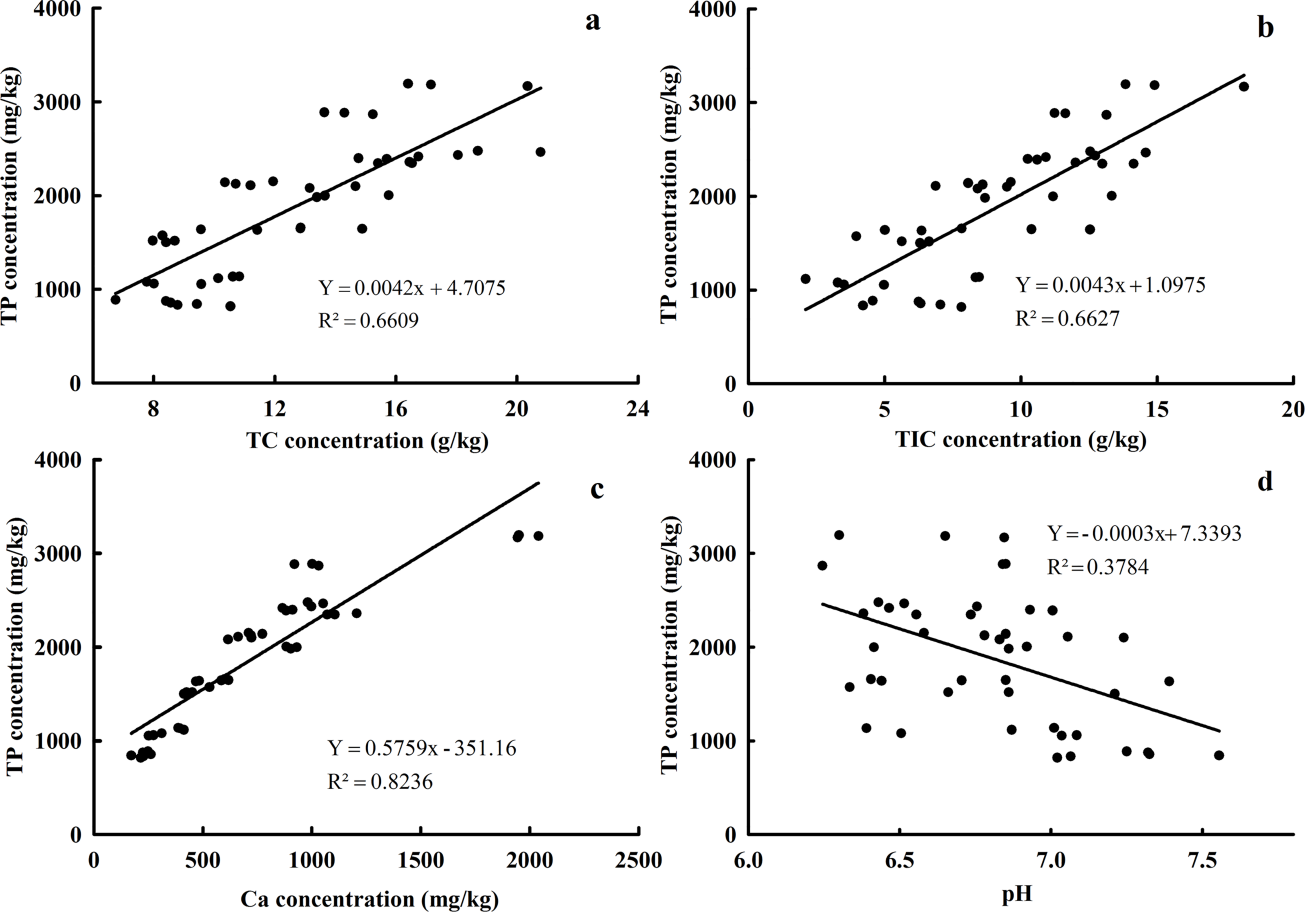
Relationship between total P and soil physicochemical properties under three irrigation patterns.

Mean TP was the highest (3180 mg P/kg soil) under FI at the depths of 0-10cm, indicating that FI favored the accumulation of TP, which may be attributed to large irrigation volume and high irrigation position. Different irrigation condition can alter soil properties. The amount of TP in soil was mostly associated with pH, TC, TIC and Ca (Fig 2). pH [42], TC [43] and calcium carbonate [44] have been reported to be closely related to the P retention ability. The content of TP was positively correlated with the levels of TC, TIC and Ca, with correlation coefficients of 0.8130, 0.8141 and 0.9075 (n = 45, p<0.01), respectively (Fig 2A, 2B and 2C). The major reason for this is that the TC and TIC contents are affected by soil organic matter [45]. Luo [46] found the significantly positive correlation between TP and soil organic carbon contents. The highly positive correlation presented between TP and extractable Ca (Fig 2C) indicates Ca plays an important role in the P accumulation [44]. TP content increased with the increasing Ca content into soil, and the possible reason is Ca ions are released into solution through ion exchange reactions, and readily combine with phosphate to form insoluble compounds [47–49]. Kleinman [50] also found a significant relationship between soil P accumulation and Ca in a group of alkaline soils. However, the negative relationship was observed between TP and pH value in our study. In most neutral soils, a pH decrease will favor the P accumulation, as retention and precipitation processes in which iron and aluminum oxides are involved are normally more efficient at lower pH [51]. In addition, a long-term FI with the highest irrigation volume (383 m^3^/ha) may result in soil harden, and restrict the mobilization and transport of P into soil profiles. In contrast, the changes of TP contents indicated that SUR led to a great accumulation of P pool in 30–60 cm layer, mainly because the irrigation amount and irrigation frequency under SUR may accelerate P vertical movement, which led to substantial P accumulation in the bottom soil layer. Significant differences were found among three treatments at depth of 0–60 cm.

#### Available P

**Fig 3:**
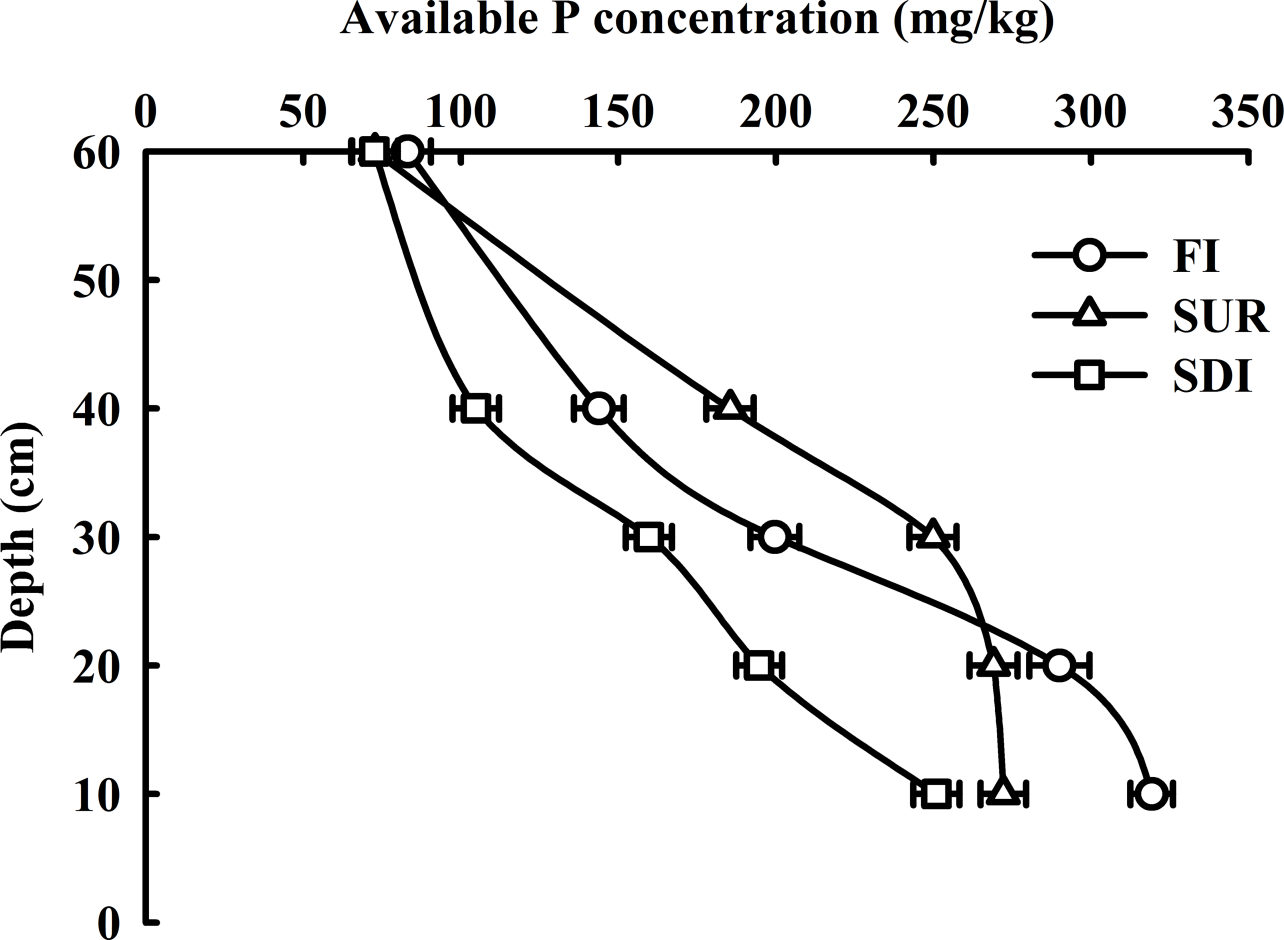
AP distribution in soil profile (0–60 cm).

The distributions of AP under three treatments in soil profiles are given in Fig 3. Generally, the contents of available P were 72.6 to 319.3 mg P/kg soil through soil profiles. In the 0–20 cm and 40–60 cm soil layers, AP contents followed the order FI>SUR>SDI, whereas AP content under SUR was slightly higher than that under SDI and FI at 30–40 cm soil depths, with the most significant difference found in this horizon under three irrigation patterns.

The value of AP in the topsoil tended to be higher under FI than those under SUR and SDI. This indicated that AP movement was limited to the surface horizons under high soil moisture and poor aeration conditions in FI. However, the content of available P under SUR was the highest in 30–40 cm soil layers, and it was mainly because the irrigation amount (174 m^3^/ha) and irrigation frequency of SUR may favor the P vertical movement and accumulation. Yang [44] reported similar observations that P amount increased in drip irrigation compared to control treatment. Significant differences were found among three treatments at depth of 0–60 cm.

#### P forms

Table 2 summarized the content and fraction distribution of P and the relative contributions of each fraction to TP under three irrigation patterns.

**Table 2:** The concentration and proportion of P fractions in soil profiles under three irrigation patterns.

Soil	Methods	Soil P forms (mg/kg)							
Depth (cm)		H_2_O-P_i_	H_2_O-P_o_	NaHCO_3_-P_i_	NaHCO_3_-P_o_	NaOH-P_i_	NaOH-P_o_	HCl-P	Residual-P
**0**–**10**	**FI**^A^	56.2a (1.77)	47.1a (1.48)	468.2a (14.72)	184.3a (5.79)	792.6a (24.92)	277.1a (8.71)	972.3a (30.57)	382.4a (12.03)
	**SUR**^B^	51.4a (2.09)	38.3b (1.56)	374.5b (15.25)	151.6b (6.17)	642.0b (26.14)	213.2b (8.68)	710.3b (28.92)	275.1b (11.2)
	**SDI**^C^	42.0b (1.79)	27.3c (1.16)	335.6c (14.29)	148.7b (6.33)	574.9c (24.48)	203.0b (8.64)	789.0c (33.59)	228.2c (9.72)
**10**–**20**	**FI**	48.1a (1.67)	35.9a (1.25)	416.1a (14.46)	167.1a (5.81)	721.5a (25.06)	236.4a (8.21)	921.4a (32.01)	331.9a (11.53)
	**SUR**	49.9a (2.08)	31.6a (1.32)	361.6b (15.07)	142.8b (5.95)	633.8b (26.41)	208.2b (8.68)	708.0b (29.5)	264.1b (11)
	**SDI**	40.5b (2.03)	23.0b (1.15)	290.2c (14.55)	137.1b (6.83)	470.3c (23.59)	166.7c (8.36)	703.0b (35.26)	164.1c (8.23)
**20**–**30**	**FI**	34.9a (1.63)	24.0ab (1.17)	416.1a (12.81)	128.0a (5.99)	509.2a (23.85)	181.6a (8.51)	726.2a (34.01)	256.9a (12.03)
	**SUR**	37.0a (1.76)	25.4a (1.21)	361.6b (15.44)	105.2b (5.02)	545.3b (26.02)	180.7a (8.62)	645.5b (30.8)	233.2b (11.13)
	**SDI**	35.7a (2.33)	18.9b (1.15)	290.2c (13.03)	71.5c (4.34)	401.9c (24.39)	128.8b (7.81)	638.2b (38.72)	135.7c (8.23)
**30**–**40**	**FI**	29.5a (1.95)	20.0a (1.32)	248.4a (16.45)	94.6a (6.26)	296.4a (19.69)	138.3a (9.16)	505.4a (33.46)	176.8a (11.71)
	**SUR**	25.1a (1.55)	17.2ab (1.07)	277.3a (17.18)	85.4a (5.29)	359.1b (22.25)	126.5a (7.84)	542.6b (33.62)	180.5a (11.19)
	**SDI**	28.8a (2.56)	14.6b (1.29)	109.1b (9.67)	38.2b (3.39)	219.2c (19.43)	95.3b (8.45)	428.2c (37.95)	194.8a (17.27)
**40**–**60**	**FI**	14.5b (1.67)	11.1a (1.27)	102.7a (b (11.79)	60.7a (6.97)	114.5a (13.13)	70.5a (8.09)	361.3a (41.45)	136.2a (15.63)
	**SUR**	12.3b (1.16)	8.1a (0.76)	133.6a (12.58)	69.5a (6.54)	150.7b (14.18)	92.5b (8.71)	454.0b (42.74)	140.5a (13.32)
	**SDI**	24.2a (2.92)	10.5a (1.27)	69.3b (8.11)	20.7b (2.5)	174.5c (21.02)	54.0a (6.51)	352.8a (42.5)	126.0a (15.18)

Different lowercase letters indicate a significant difference at P <0.05 between treatments in each soil horizon.

Values in parenthesis represent proportion (%) of TP in soil.

A furrow irrigation.

B surface drip irrigation.

C subsurface drip irrigation.

The H_2_O-P concentration declined with increasing depth. The content of organic P forms with H_2_O-P_o_ ranged 12.3–56.2 mg/kg. Among the sequentially extracted P forms, the content of H_2_O-P accounted for 4% of the total in the soil profiles under FI, SUR, and SDI, respectively. The greatest proportion of H_2_O-P was observed in all soil layers under SUR. The majority of the P in the NaHCO_3_ fraction was observed in the inorganic formranging from 69.3 to 468.2 mg/kg. The NaHCO_3_-P_i_ fraction ranged from about 11 to 16% under FI, 12 to 17% under SUR and 8 to 14% under SDI. The highest proportion of NaHCO_3_-P_i_ were detected in all soil layers under SUR. The NaHCO_3_-P_o_ fraction was lower than NaHCO_3_-P_i_ in all soil layers under all three irrigations (Table 2). Among the three treatments, NaOH-P_i_ values decreased with soil depths within 0–60 cm, and clear discrepancies were exhibited under three methods (Table 2). NaOH-P_i_ proportion to TP ranged from 14.2 to 24.9% which is more than that reported by Adhami in neutral soil (2–6%) [52]. The portion of NaOH-P_i_ in soil profiles under SUR was higher than those under FI and SDI. The significant differences on the portion of NaOH-P_i_ were observed in all soil layers among three treatments. Under three irrigation patterns, the NaOH-P_o_ value to TP remained around 8% without significant change with different depths and irrigation patterns. The proportion of NaOH-P_o_ in SUR was higher than the other two treatments in 0–30 cm and 40–60 cm soil horizons. However, the value of NaOH-P_o_ was greater under FI than those under SUR and SDI in 30–40 cm. HCl-P contents and their proportion to TP under different irrigations are shown in Table 1. In general, the contents of different HCl-P followed the order FI> SUR> SDI, and decreased with depth. The contents of HCl-P ranged from 352.8 to 972.3 mg/kg under three irrigations (Table 2). P extracted with HCl ranged from 31 to 41% under FI, 29 to 43% under SUR, and 34 to 43% under SDI (Table 2). The content of residual P fraction decreased with depth in this study. The residual P value ranged from 136.2 to 382.4 mg/kg under FI, 140.5 to 275.1 mg/kg under SUR, and 126.0 to 228.2 mg/kg under SDI. The proportion of residual P varied from 12to 15% under FI, 11 to 13% under SUR, and % to 15% under SDI. The proportion of residual P under FI was higher than those under SUR and SDI in 0–30 cm and 40–60 cm soil layers. However, a higher proportion of residual P was observed under SDI compare with FI and SUR at 30–40 soil layer.

Different irrigation patterns can alter soil properties, thus affecting the transformation and movement of soil nutrients, and then soil P fractions [32]. The concentration of P in eight fractions were tested for correlations with soil properties, such as pH, TC, TOC, TIC, Fe_ox_, Al_ox_, and Ca under the three irrigation patterns. Correlation coefficients were used to characterize the relationship between P fractions and soil properties, as shown in Table 3. The contents of various forms of P were significantly and positively correlated with the levels of their corresponding solid phases. All forms of P were significantly positively correlated with the TC, TIC, and Ca contents, although negatively correlated with the pH (in Pearson’s correlation coefficients in Table 3). The negative relationship between NaOH-P and pH has already been highlighted by Adhami [52]. HCl-P was the predominant form of P in all three treatments. HCl-P was extremely linked to Ca compounds [0.902, (n = 45)], which is consistent with previous studies [53–57]. The major reason for this is the high concentrations of Ca compounds led to the chemical reaction between released P from primary apatite P and calcium minerals under neutral or alkaline conditions [58], which implied that most of the P was mainly precipitated as calcium phosphates or precipitates with carbonates [59]. This is consistent with the results reported by Adhami who observed a significantly positive correlation between calcium carbonate and HCl-P.

**Table 3:** The correlation coefficient between the P forms and soil properties.

Soil	Soil P forms							
Properties	H_2_O-P_i_	H_2_O-P_o_	NaHCO_3_-Pi	NaHCO_3_-P_o_	NaOH-P_i_	NaOH-P_o_	HCl-P	Residual-P
**TC**	0.860**	0.832**	0.759**	0.753**	0.841**	0.793**	0.777**	0.677**
**TOC**	0.067	0.015	0.05	−0.08	0.052	0	−0.107	−0.03
**TIC**	0.827**	0.821**	0.735**	0.782**	0.814**	0.788**	0.816**	0.686**
**Fe**_**ox**_	0.064	−0.125	−0.087	−0.16	−0.012	−0.109	−0.08	−0.322*
**Al**_**ox**_	0.301*	0.183	0.147	0.168	0.245	0.167	0.219	−0.058
**Ca**^2+^	0.833**	0.892**	0.854**	0.855**	0.881**	0.900**	0.902**	0.837**
**pH**	−0.688**	−0.616*	−0.613**	−0.552**	−0.629**	−0.637**	−0.571**	−0.518**

**Significant at P<0.01.

*Significant at P<0.05.

Soil P was separated into the following: water-soluble P (H_2_O-P_i_ and H_2_O-P_o_), labile P (NaHCO_3_-P_i_ and NaHCO_3_-P_o_), moderately labile P (NaOH-P_i_ and NaOH-P_o_), moderately resistant P (HCl-P), and residual P. Water-soluble P and labile P were considered “readily mineralizable” and significantly related to P uptake by plants [60]. The total contents of water-soluble P and labile P were greater under FI (603 to 755.8 mg/kg) than those under SUR (529.2 to 615.8 mg/kg) and SDI (416.3 to 553.6 mg/kg) in 0–30 cm soil layers. The reason may be different irrigation schedules influenced soil moisture and aeration conditions. The largest irrigation amount was used under FI under the upper horizons, resulting in high evaporation reaction and low soil water infiltration. This indicated the mobilization and transport of H_2_O-P and NaHCO_3_-P were restricted in the topsoil layers. However, the total contents of H_2_O-P and NaHCO_3_-P in lower layers (30–60 cm) were higher under SUR than those under the other two irrigation treatments. The results may be attributed to appropriate soil moisture content under SUR facilitated the movement of H_2_O-P and NaHCO_3_-P to deeper soil layers with soil water infiltration.

NaOH-P was associated with Fe and Al oxides and considered less available to plants [61–62]. However, NaOH-P were significantly positively correlated with the TC, TIC, and Ca contents in our study (Table 3). The contents of NaOH-P in the upper soil layers was greater under FI than those under SUR and SDI, indicating the NaOH-P concentration increased with the increasing contents of carbon and carbonate.

HCl-P was considered bound with Ca in carbonate or phosphate minerals. The proportion of HCl-P to TP under SUR was the lowest, and it was mainly attributed to the Ca contents which was less than those under FI and SDI in soil profiles. The result showed that HCl-P was a principal form, because it was bound to Ca whose value was up to 205 to 2378 mg/kg in all three irrigation patterns in soil profiles. The amount and percentage of this fraction was obviously higher than other P forms. This result is consistent with the result from previous study that showed HCl-P fraction is the most dominant fraction in all soils [63]. Irrigation patterns significantly affected HCl-P concentration at the depth of 0 to 10 cm and 30 to 40 cm due to the extra variation of soil moisture.

### The availability of P fractions under different irrigations

The relation between the available P and the P forms of soil was established using the field study results (Table 4). Based on the above values, the coefficients of determination for linear regression between available P and P fractions among three treatments were found significant. The results showed that NaHCO_3_-P_o_ under FI is an important factor in determining the amount of available P. The H_2_O-P_o_, NaHCO_3_-P_o_, and NaOH-P_i_ contents were considered the main factors that influenced the available P values under SUR, respectively. AP correlated significantly with the contents of NaHCO_3_-P_o_ and NaOH-P_o_ under SDI.

**Table 4:** Regression models describing the relationship between the AP (y) and the forms of P (x).

Treatment	Regression Models	Coefficient data	R^2^	
FI^A^	y = a+ bx_2_	a = -38.73, b = 1.937	0.997**	
SUR^B^	y = a+ b_1_x_3_+ b_2_x_2_+ b_3_x_1_	a = 79.486, b_1_ = 0.461, b_2_ = - 1.327, b_3_ = 2.591	0.995**	
SDI^C^	y = a+ b_1_x_4_+ b_2_x_3_	a = 9.776, b_1_ = 0.615, b_2_ = 0.179	0.992**	

x_1_: H_2_O-P_o_, x_2_: NaHCO_3_-P_o_, x_3_: NaOH-P_i_, X_4_: NaOH-P_o_

**Significant at P<0.01. *Significant at P<0.05.

A furrow irrigation.

B surface drip irrigation.

C subsurface drip irrigation.

Among all three irrigation patterns, the NaHCO_3_-P_**o**_ played an important role in AP, because it is decomposed and adsorbed easily by crop. The NaOH-P_o_ and NaOH-P_i_ were the potential P resource under SUR and SDI, and the contents increased with the increase of iron and aluminum oxides.

### Influence of different irrigation patterns on yields

**Fig 4:**
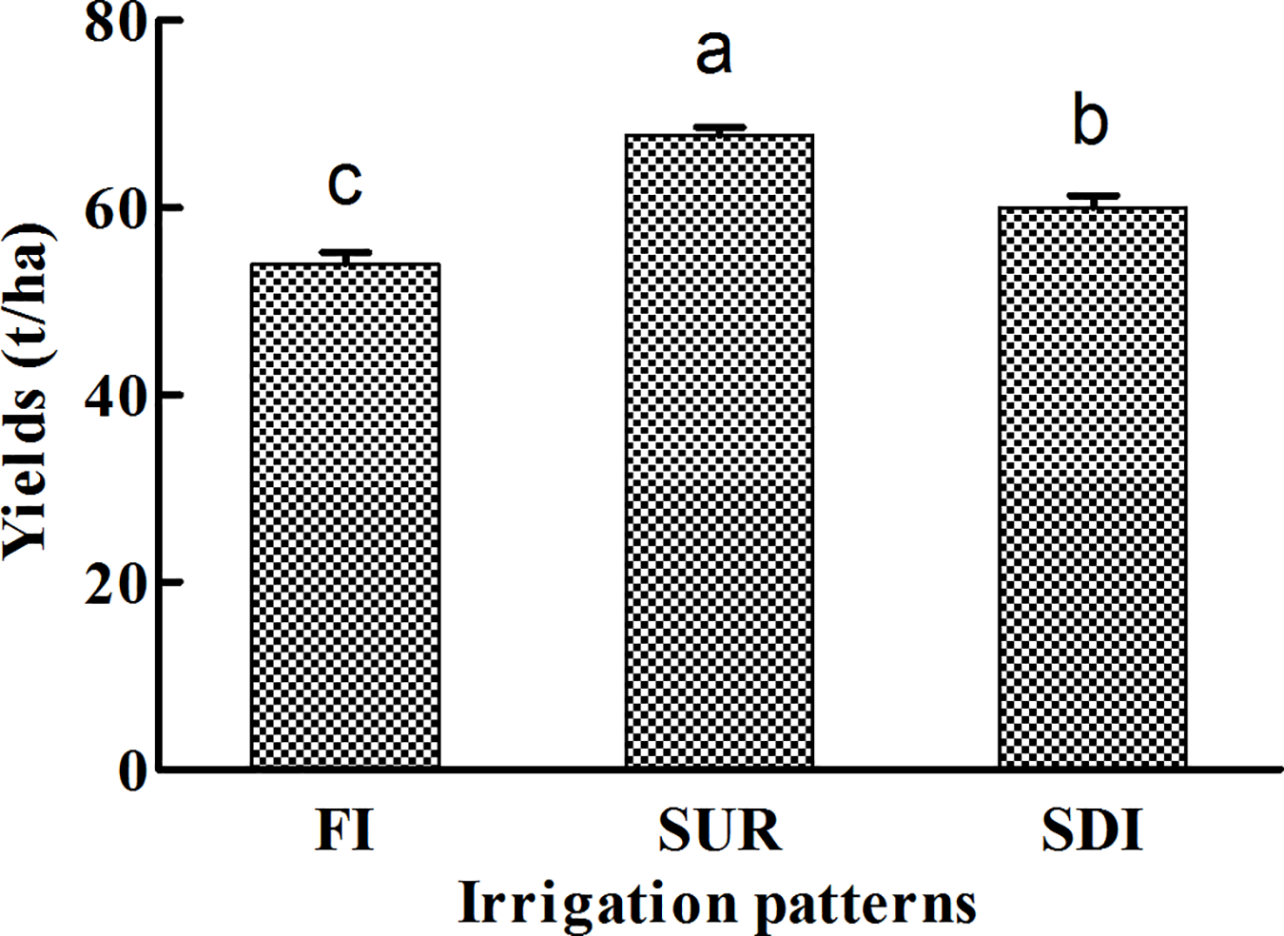
Tomato yields under three irrigation patterns. Error bars represent standard errors of the means. Bars marked by the different letter are significantly different at p<0.05.

Fig 4 showed the tomato yields under three irrigation patterns.

The yield was higher (67 t/ha) under SUR than that under SDI (60 t/ha) and FI (54 t/ha). Compared to FI, tomato yield increased by 24% under SUR and by 11% under SDI. Significant differences were found among three treatments. The main reason was SUR favored the accumulation of AP at 20 to 60 cm, which may be attributed to moderate irrigation amount and irrigation frequency. The highest yield under SUR could be related to a higher P uptake by roots.

## Conclusions

The results of this study demonstrate that the difference of long-term irrigation patterns has great effects on the P concentration and form distributions in soil.

It was found the contents of TP and AP decreased with the increasing depths under all three irrigations. FI led to greatest accumulation of TP and AP at topsoil layer, which would prevent a substantial amount of AP from being uptaken by plants. The ranking order of P fractions content was HCl-P > NaHCO_3_-P > NaOH-P > Residual-P > H_2_O-P in all the samples. The relationship between P fractions and soil properties showed all forms of P were significantly positively correlated with the TC, TIC, and Ca contents, but negatively with the pH. Under all three irrigation treatments, H_2_O-P_o_, NaHCO_3_-P_o_, and NaOH-P played key roles in AP because it could be decomposed easily and adsorbed by crop. Comparison among three irrigation patterns indicated the sum proportion of H_2_O-P_o_, NaHCO_3_-P_o_, and NaOH-P to TP was higher under SUR than those under FI and SDI in soil profiles, the result indicated SUR could easily supply AP for crop when AP was limited in this study. The tomato yield under SUR was the highest among all treatments.
